# Cardiovascular risk factors in Chiari malformation and idiopathic intracranial hypertension

**DOI:** 10.1002/brb3.677

**Published:** 2017-03-28

**Authors:** Radek Frič, Are Hugo Pripp, Per Kristian Eide

**Affiliations:** ^1^ Department of NeurosurgeryOslo University Hospital–RikshospitaletOsloNorway; ^2^ Faculty of MedicineUniversity of OsloOsloNorway; ^3^ Oslo Centre of Biostatistics and EpidemiologyOslo University HospitalOsloNorway

**Keywords:** arterial hypertension, Chiari malformation type I, diabetes, HUNT 3, idiopathic intracranial hypertension

## Abstract

**Objectives:**

Both Chiari malformation type 1 (CMI, i.e., the idiopathic caudal ectopy of cerebellar tonsils into foramen magnum) and idiopathic intracranial hypertension (IIH) are characterized by reduced intracranial compliance (ICC) due to disturbed circulation of cerebrospinal fluid (CSF). An increasing body of evidence links cardiovascular disease to CSF circulation disturbances. The aim of this study was to explore whether the prevalence of cardiovascular risk factors in patients with CMI or IIH is higher than in the general population.

**Materials and Methods:**

Among the patients with CMI or IIH treated at our department during the period 2003–2014, we identified those with history of arterial hypertension (AH), myocardial infarction (MI), angina pectoris (AP), or diabetes mellitus (DM). For comparison with a control population, we retrieved information about the prevalence of AH, MI, AP, and DM among participants of the North‐Trøndelag Health Study 3 (HUNT3).

**Results:**

Data from 48 CMI and 52 IIH cases were available. Compared to data from the 42,461 individuals participating in the HUNT3, we found increased prevalence of DM in male CMI as well as female IIH cases, and of AH in female IIH cases. Body mass index (BMI) was significantly increased in both female and male IIH cases. Prevalence of MI and AP in the CMI and IIH cohorts was extremely low and therefore not further studied.

**Conclusions:**

This study provided evidence of an increased prevalence of DM in male CMI as well as female IIH cases and of AH in female IIH cases. Although requiring further exploration, these findings point to AH and DM as potential risk factors in the pathophysiology of CMI and IIH.

## 1 Introduction

Chiari malformation type 1 (CMI), characterized by idiopathic caudal ectopy of cerebellar tonsils into foramen magnum (Milhorat et al., 1999), and idiopathic intracranial hypertension (IIH) (Friedman, Liu, & Digre, 2013) are traditionally considered to be two distinct clinical entities, although some common clinical, radiological, and pathophysiological features have been noticed (Bejjani, 2003; Frič & Eide, 2016). In particular, disturbed cerebrospinal fluid circulation (CSF) resulting in reduced intracranial compliance (ICC) has been demonstrated as a characteristic feature of both CMI and IIH (Eide & Kerty, 2011; Frič & Eide, 2015, 2016).

The recent recognition of the intracerebral paravascular (glymphatic) circulation (Iliff & Nedergaard, 2013; Jessen, Munk, Lundgaard, & Nedergaard, 2015) links the cardiovascular disease and the vascular risk factors even closer to conditions presenting with disturbed CSF circulation. It is therefore of crucial importance to improve our understanding of the relation between these conditions and the cardiovascular comorbidity.

The etiology and pathophysiology of CMI and IIH still remains unclear, including the role of cerebrovascular comorbidity. The evidence of significantly increased prevalence of cardiovascular disease and diabetes mellitus (DM) in both idiopathic normal pressure hydrocephalus (iNPH) (Eide & Pripp, 2014) and noncommunicating hydrocephalus (ncHC) (Eide & Pripp, 2016) has been provided, suggesting that cardiovascular disease and DM is involved as an exposure factor in the development of both communicating and noncommunicating forms of hydrocephalus. Accordingly, we asked therefore whether a similar association might be found in patients with CMI or IIH.

The purpose of this study was to examine whether the occurrence of cardiovascular risk factors in CMI and IIH patients is higher than in the general population. We hypothesized that there should be a significant association between prevalence of the cardiovascular disease and DM in patients with CMI or IIH. To the best of our knowledge, this issue has not been specifically addressed previously.

## 2 Methods

### 2.1 Patient material

The study was approved by The Regional Committee for Medical and Health Research Ethics (REK) of Health Region South‐East, Norway (2012/1180), and by Oslo University Hospital (2011/6692), Oslo, Norway.

The patient material included the patients with CMI and IIH referred to treatment at the Department of neurosurgery, Oslo University Hospital, Rikshospitalet, during the 12‐year period from 2003 to 2014. As practically all patients with CMI and IIH underwent invasive measurement of intracranial pressure (ICP) as a part of diagnostic work‐up in this period, we identified them retrospectively from the department's database of ICP measurements. For the purpose of this study, we included only patients older than 20 years at the time of presentation.

The diagnosis of CMI was based on magnetic resonance (MRI) findings of significant ectopy of cerebellar tonsils (i.e., ≥5 mm below the level of foramen magnum), with or without associated syringomyelia, and symptoms related to these findings.

The diagnosis of IIH was based on a typical history and symptoms, ophthalmological findings, increased opening pressure of cerebrospinal fluid (CSF) during lumbar puncture, and in some cases MRI findings typical (but not obligatory) for the diagnosis (empty sella, flattening of the posterior aspect of the ocular globe, distension of the perioptic subarachnoid space with or without a tortuous optic nerve, and transverse venous sinus stenosis). In addition, intracranial hypertension was verified by invasive ICP measurement.

### 2.2 General population

The prevalence of cardiovascular disease and DM in a general population was estimated from Nord‐Trøndelag Health Study 3 (HUNT3, http://www.ntnu.no/hunt). In this population‐based Norwegian public health study that has run since 1984, the inhabitants of the county of Nord‐Trøndelag, Norway, aged 20 years and older were invited to participate in a general health survey that also included a questionnaire on cardiovascular disease and DM. More than 50,000 individuals participated in HUNT3 (2006–2008) survey. The population of Nord‐Trøndelag County is stable and homogenous with <3% of non‐Caucasians, and may be considered representative for Norway in general, though not containing any larger cities.

### 2.3 Prevalence of cardiovascular disease and diabetes

Cardiovascular disease and DM in CMI and IIH patients was reported by the referring general practitioner or neurologist, and/or by the patient or his/her relatives at the time of admission to our department. The HUNT3 used a standardized questionnaire for the participants where the occurrence of cardiovascular disease and DM was self‐reported by answering the following questions:

- Do you take or have you taken medication for high blood pressure ?
- Have you had or do you have any of the following: angina pectoris (chest pain) ?
- Have you had or do you have any of the following: myocardial infarction (heart attack) ?
- Have you had or do you have any of the following: diabetes ?

### 2.4 Prevalence of cardiovascular disease and diabetes versus pulsatile intracranial pressure

The CMI and IIH patients included in this study had undergone overnight monitoring of pulsatile ICP, as previously described (Eide & Sorteberg, 2010; Frič & Eide, 2016). We dichotomized patients according to threshold for abnormal pulsatile ICP, that is, mean ICP wave amplitude (MWA) >4 mmHg on average during an overnight monitoring, and >5 mmHg during more than 10% of the recording time (Eide & Sorteberg, 2010). The prevalence of cardiovascular disease and DM in patients with MWA either below or above these thresholds was determined.

### 2.5 Statistical analysis

Statistical analyses were performed using the SPSS software version 22 (IBM Corporation, Armonk, NY, USA; RRID:SCR_002865) and Stata version 13 (StataCorp LP, College Station, TX, USA; RRID:SCR_01276). Descriptive statistics are mean (standard deviation) or number of patients (percentage). Difference between groups was assessed with Student *t* test or chi‐square tests for crosstabs if not otherwise stated. Odds ratios (OR) with 95% confidence intervals (95 CI) and *p*‐values were calculated using logistic regression analysis. To take into account both the confounding and modifying effects of gender, stratified analysis on gender was conducted. To adjust for any confounding effect by differences in age distribution between cases and controls, logistic regression with age as a continuous independent variable was also performed. Statistical significance was accepted at the 0.05 level.

## 3 Results

### 3.1 Patients

Table 1 shows demographic data from 48 CMI and 52 IIH cases, as well as from 42,461 controls participating in the HUNT3. Specifically, mean age in both patient cohorts was equal and significantly lower than in the HUNT3 cohort. Moreover, both CMI and IIH cohorts were characterized by significant female predominance.

**Table 1:** Demographic data of CMI and IIH cases and HUNT3 controls

	Case cohorts		Control cohort	
	CMI	IIH	HUNT3	*p*‐value
*N*	48	52	42,461	
Gender (F/M)	35/13	43/9	23,150/19,311	**<.001**
Mean age (±SD)	35.3 ± 11.6	35.3 ± 10.4	48.7 ± 12.9	**<.001**
BMI (kg/m^2^)	27.4 ± 6.6	32.4 ± 6.3	27.1 ± 4.4	**<.001**
Arterial hypertension (N/%)	3 (6.3)	7 (13.5)	6,597 (15.4)	.190
Diabetes mellitus (N/%)	2 (4.1)	5 (9.6)	1,453 (3.4)	**.048**
Cardiac infarction (N/%)	0 (0)	0 (0)	749 (1.8)	.407
Angina pectoris (N/%)	0 (0)	1 (1.9)	745 (1.8)	.649

CMI, Chiari malformation type I; IIH, idiopathic intracranial hypertension; HUNT3, The HUNT3 Survey.

Assessment of statistical significance: independent samples *t* test for continuous data and Pearson chi‐square test for categorical data.

Taken CMI and IIH cohorts together and in the comparison to the HUNT3 cohort, the prevalence of DM was slightly significantly increased (*p *= .048) while prevalence of AH was increased only insignificantly (*p *= .19). The occurrence of angina pectoris (AP) and myocardial infarction (MI) in the CMI and IIH cohorts was so low that it was not further studied.

The body mass index (BMI; defined as body weight in [kg] divided by the square of body height in [m]) was significantly different between groups (*p *< .001; Table 1). Gender‐ and age‐adjusted analysis revealed a significantly increased BMI in both female and male IIH patients (*p *< .001 and *p *= .018; Table 2). Also in female CMI cases, the BMI was close to significantly increased (*p *= .057; Table 2).

**Table 2:** BMI in CMI and IIH cases versus HUNT3 controls adjusted according to gender and age

	Gender	Total	BMI Mean (SD)	Crude estimate	Age‐adjusted estimate
				OR (95 CI), *p*‐value	OR (95% CI), *p*‐value
CMI	Female	35	27.7 (7.2)	1.03 (0.96–1.11), *p *= .360	1.06 (1.00–1.13), *p *= .057
IIH	Female	43	32.9 (6.6)	1.20 (1.15–1.26), *p *< .001	1.21 (1.16–1.26), ***p *** **< .001**
HUNT3	Female	23,020	27.1 (4.4)	Control group	Control group
CMI	Male	13	26.6 (4.8)	0.97 (0.84–1.10), *p *= .610	1.03 (0.92–1.15), *p *= .590
IIH	Male	9	30.0 (4.0)	1.11 (1.00–1.25), *p *= .06	1.13 (1.02–1.25), ***p *** **= .018**
HUNT3	Male	19,202	27.2 (4.5)	Control group	Control group

BMI, Body mass index; CMI, Chiari malformation type I; IIH, idiopathic intracranial hypertension; HUNT3, The HUNT3 Survey; OR, odds ratio; CI, confidence interval.

Data presented as numbers (percentages in parentheses) and statistically assessed with logistic regression analysis.

### 3.2 Prevalence of arterial hypertension and diabetes in CMI and IIH patients

In a detailed analysis, the prevalence of AH was significantly increased (*p *= .015) in female IIH cases as compared to the general population (Table 3). This was a case prevalent neither in male IIH patients nor in female/male CMI cases.

**Table 3:** Prevalence of arterial hypertension according to gender and age in CMI and IIH cases as compared to the HUNT3 controls

	Gender	Total	Arterial hypertension		Crude estimate	Age‐adjusted estimate
			Yes (%)	No (%)	OR (95% CI), *p*‐value	OR (95% CI), *p*‐value
CMI	Female	35	2 (5.7)	33 (94.3)	0.35 (0.08–1.46), *p *= .149	0.85 (0.19–3.77), *p *= .830
IIH	Female	43	6 (14.0)	37 (86.0)	0.93 (0.40–2.21), *p *= .887	3.27 (1.25–8.51), ***p *** **= .015**
HUNT3	Female	23,150	3,424 (14.8)	19,726 (85.2)	Control group	Control group
CMI	Male	13	1 (7.7)	12 (92.3)	0.42 (0.06–3.26), *p *= .410	3.65 (0.38–35.2), *p *= .263
IIH	Male	9	1 (11.1)	8 (88.9)	0.64 (0.08–5.08), *p *= .669	2.51 (0.25–25.5), *p *= .438
HUNT3	Male	19,311	3,173 (16.4)	16,138 (83.6)	Control group	Control group

CMI, Chiari malformation type I; IIH, idiopathic intracranial hypertension; HUNT3, The HUNT3 Survey; OR, odds ratio; CI, confidence interval.

Data presented as numbers (percentages in parentheses) and statistically assessed with logistic regression analysis.

The prevalence of DM was significantly increased (*p *= .045) in male CMI cases and highly significantly increased (*p *< .0001) in female IIH cases, as compared to the general population (Table 4).

**Table 4:** Prevalence of diabetes according to gender and age in CMI and IIH cases as compared to the HUNT3 controls

	Gender	Total	Diabetes		Crude estimate	Age‐adjusted estimate
			Yes (%)	No (%)	OR (95% CI), *p*‐value	OR (95% CI), *p*‐value
CMI	Female	35	1 (2.9)	34 (97.1)	0.99 (0.14–7.28), *p *= .996	1.89 (0.25–14.1), *p *= .535
IIH	Female	43	5 (11.6)	38 (88.4)	4.45 (1.75–11.3), *p *= .002	9.94 (3.77–26.3), ***p *** **< .001**
HUNT3	Female	23,150	665 (2.9)	22,485 (97.1)	Control group	Control group
CMI	Male	13	1 (7.7)	12 (92.3)	1.96 (0.25–15.1), *p *= .519	8.71 (1.05–72.0), ***p *** **= .045**
IIH	Male	9	0 (0)	9 (100)	Not defined	Not defined
HUNT3	Male	19,311	788 (4.1)	18,523 (95.9)	Control group	Control group

CMI, Chiari malformation type I; IIH, idiopathic intracranial hypertension; HUNT3, The HUNT3 Survey; OR, odds ratio; CI, confidence interval.

Data presented as numbers (percentages in parenthesis) and statistically assessed with logistic regression analysis.

### 3.3 Prevalence of arterial hypertension and diabetes for different levels of pulsatile ICP

Comparing patients with MWA levels below/above the threshold for abnormality, the prevalence of AH was significantly increased (*p *= .003) in IIH cases with abnormal MWA (i.e., above the threshold), while the prevalence of DM was significantly increased (*p *< .001) in IIH cases with MWA below the threshold (Table 5).

**Table 5:** Prevalence of arterial hypertension and diabetes according to MWA levels in CMI and IIH patients

	Total	Comorbidity		Crude estimate	Age‐adjusted estimate
		Yes (%)	No (%)	OR (95% CI), *p*‐value	OR (95% CI), *p*‐value
Arterial hypertension					
CMI: MWA <4 mmHg	12	1 (8.3)	11 (91.7)	0.49 (0.06–3.83), *p *= .500	1.77 (0.20–16.1), *p *= .610
CMI: MWA ≥4 mmHg	36	2 (5.6)	34 (94.4)	0.32 (0.08–1.33), *p *= .117	1.01 (0.23–4.54), *p *= .987
HUNT3	42,461	6,597 (15.5)	35,864 (84.5)	Control group	Control group
IIH: MWA <4 mmHg	8	0 (0)	8 (100)	Not defined	Not defined
IIH: MWA ≥4 mmHg	44	7 (15.9)	37 (84.1)	1.02 (0.46–2.31), *p *= .946	4.11 (1.64–10.3), ***p *** **= .003**
HUNT3	42,461	6,597 (15.5)	35,864 (84.5)	Control group	Control group
Diabetes					
CMI: MWA <4 mmHg	12	0 (0)	12 (100)	Not defined	Not defined
CMI: MWA ≥4 mmHg	36	2 (5.6)	34 (94.4)	1.66 (0.40–6.92), *p *= .486	4.02 (0.94–17.3), *p *= .061
HUNT3	42,461	1,453 (3.4)	41,008 (96.6)	Control group	Control group
IIH: MWA <4 mmHg	8	3 (37.5)	5 (62.5)	16.9 (4.0–70.9), *p *< .001	50.2 (10.8–232.5), ***p *** **< .001**
IIH: MWA ≥4 mmHg	44	2 (4.6)	42 (95.4)	1.34 (0.33–5.56), *p *= .683	3.24 (0.76–13.8), *p *= .111
HUNT3	42,461	1,453 (3.4)	41,008 (96.6)	Control group	Control group

CMI, Chiari malformation type I; MWA, mean wave amplitude; HUNT3, The HUNT3 Survey; IIH, idiopathic intracranial hypertension; OR, odds ratio; CI, confidence interval.

Data presented as numbers (percentages in parenthesis) and statistically assessed with logistic regression analysis.

## 4 Discussion

The main finding from this study was the increased prevalence of diabetes in male CMI and female IIH cases as well as the increased prevalence of arterial hypertension in female IIH cases. Moreover, the prevalence of AH was increased in IIH cases with abnormal pulsatile ICP, whereas prevalence of DM was increased in IIH cases with pulsatile ICP below the threshold for abnormality.

### 4.1 The pathophysiology of CMI and IIH

The etiology of both conditions remains poorly understood. There is also still a limited understanding of pathophysiology behind both CMI and IIH. The elevated pulsatile intracranial pressure (ICP), indicative of reduced intracranial compliance (ICC), has been documented in treatment‐responsive individuals with CMI (Frič & Eide, 2015) or IIH (Eide & Kerty, 2011; Frič & Eide, 2016). The reduced ICC is a result of the disturbed cerebrospinal (CSF) circulation that has changed the relation between intracranial pressure and volume, that is, the pressure–volume reserve capacity (Eide, 2016). This links both CMI and IIH to conditions like communicating, denoted idiopathic normal pressure hydrocephalus (iNPH), and noncommunicating hydrocephalus (ncHC) where elevated pulsatile pressure has also been documented (Eide & Sorteberg, 2010, 2016; Saehle & Eide, 2015a, 2015b). Moreover, in all these conditions the pulsatile ICP may also be abnormally elevated due to the reduced vascular compliance resulting from the cardiovascular disease.

For the part of CMI, it would be natural to speculate that the primary reason for the disturbed CSF circulation is the obstruction of CSF flow at the level of foramen magnum due to the ectopy of cerebellar tonsils, which is the characteristic feature of CMI. However, there is no significant association between the extent of tonsillar ectopy or any other radiological features and the ICP parameters (Frič & Eide, 2015, 2016), a fact suggesting a possible role of other factors contributing to the reduced ICC in CMI.

As far as IIH is concerned, the primary reason to the reduced ICC has been believed to be an increased venous pressure in typically overweight patients. However, brain tissue expansion due to astrogliosis has been suggested as another possible contributing factor to the reduced ICC seen in IIH patients (Eide, Eidsvaag, Nagelhus, & Hansson, 2016). In the same study, increased perivascular aquaporin‐4 was demonstrated, raising the questions of the role of the paravascular interstitial (glymphatic) intracerebral circulation (Iliff & Nedergaard, 2013; Iliff et al., 2012) and the role of the microvascular changes induced by DM (Johnson, Brendel, & Meezan, 1982) with regard to the reduced ICC (Onodera, Oshio, Uchida, Tanaka, & Hashimoto, 2012). It is reasonable to believe that also systemic AH contributes to microvascular changes in the brain, although this issue has not yet been studied specifically in relation to the ICC.

### 4.2 The role of BMI

We found a significantly increased BMI in both female and male IIH cases. This complies with a well‐known fact that being overweight is a typical feature of patients with IIH (Ball & Clarke, 2006; Giuseffi, Wall, Siegel, & Rojas, 1991; Wall et al., 2014). At the same time, being overweight is a predisposition for both arterial hypertension as well as DM. An increased prevalence of AH and DM in female IIH cases therefore cannot be surprising. However, AH was significantly associated with IIH also in the study of Giuseffi et al. (1991), but not when controlling for obesity.

That increased prevalence of AH and DM was not found among male IIH cases may easily be explained by a small proportion of men in our IIH cohort (9 out of 52 cases). Female predominance is also a typical feature of IIH (Giuseffi et al., 1991; Wall et al., 2014).

On the contrary, increased prevalence of DM among male CMI cases is more difficult to explain, as their BMI actually was lower than in both female CMI cases and general population.

### 4.3 The role of arterial hypertension and diabetes in CSF circulation disorders

Only a very few studies, incorporating small numbers of patients and hospital‐based control groups, have explored the prevalence of cardiovascular disease in clinical conditions characterized by a disturbance of CSF circulation. Thus, an association between cardiovascular disease and idiopathic normal pressure hydrocephalus (iNPH) was suggested (Casmiro et al., 1989; Earnest, Fahn, Karp, & Rowland, 1974; Edwards, Dombrowski, Luciano, & Pople, 2004; Graff‐Radford & Godersky, 1987; Jaraj et al., 2016; Koto, Rosenberg, Zingesser, Horoupian, & Katzman, 1977; Krauss et al., 1996). More recently, an increased age‐adjusted prevalence of cardiovascular disease and diabetes was reported in iNPH (Eide & Pripp, 2014) and noncommunicating hydrocephalus (ncHC) (Eide & Pripp, 2016) as compared to the general population (Table 6).

**Table 6:** Summary of significantly increased prevalence of arterial hypertension and diabetes in some CSF circulation disorders according to gender and age

	Arterial hypertension		Diabetes		References
	Female	Male	Female	Male	
NPH	–	↑	↑	↑	Eide and Pripp (2014)
Noncommunicating HC	–	↑	↑	–	Eide and Pripp (2016
CMI	–	–	–	↑	Present study
IIH	↑	–	↑	–	Present study

NPH, idiopathic normal pressure hydrocephalus; noncommunicating HC, noncommunicating hydrocephalus; CMI, Chiari malformation type I; IIH, idiopathic intracranial hypertension.

To the best of our knowledge, a similar association has previously not been studied for CMI and only scarcely for IIH (Giuseffi et al., 1991). In our view, these two conditions – although each with its typical clinical and radiological characteristics (Friedman et al., 2013; Milhorat et al., 1999) – are both often characterized by some degree of reduced ICC as recently addressed (Frič & Eide, 2016). A particular aspect of these two conditions is that they are often present during early adulthood, that is, in much younger age than, for example, a typical case of iNPH. The prevalence of cardiovascular disease as well as of DM in patients with CMI and IIH (mean age 35.3 years in our study) should therefore in principle not exceed the rates from general population (mean age 48.7 years in the HUNT3 cohort). While both AH and DM may be related to being overweight in IIH patients as discussed here, we do not have such obvious explanation for the increased prevalence of DM in male CMI patients.

The CSF compartments and cerebrovascular system are closely linked (Brinker, Stopa, Morrison, & Klinge, 2014). As mentioned above, much attention has recently been paid to the role of the paravascular cerebrospinal/interstitial fluid exchange in the brain (Iliff & Nedergaard, 2013; Iliff et al., 2012; Xie et al., 2013). A possible role of disrupted paravascular flow as a key mechanism behind the elevated pulsatile ICP observed in patients with iNPH has been suggested (Eide & Sorteberg, 2016). Here, it is reasonable to speculate that diseases affecting the cardiovascular system and in particular the cerebral vasculature also influence these paravascular flow mechanisms, thus contributing to the development of disturbed CSF circulation and hence the reduced ICC.

### 4.4 Arterial hypertension and diabetes versus different levels of pulsatile ICP

Our finding of a significantly increased prevalence of AH in IIH cases with documented elevated pulsatile ICP suggests that IIH patients with AH are more likely to develop reduced ICC, that is, AH is a risk factor in the pathophysiology of the disease. Previously, it has been found an increased prevalence of arterial hypertension and diabetes also in patients with iNPH and elevated pulsatile ICP, when compared with the participants of the HUNT3 Survey (Eide & Pripp, 2016). However, in patients with ncHC and elevated pulsatile ICP, only an increased prevalence of diabetes was found, but not arterial hypertension or cardiac infarction (Eide & Pripp, 2016) (Table 6).

On the other hand, the prevalence of DM was significantly increased in our IIH cases with MWA actually below the threshold for abnormality, for which we do not find any obvious explanation. Similarly, among those iNPH patients with normal pulsatile ICP in the study of Eide and Pripp (2016), the prevalence of arterial hypertension, cardiac infarction, and diabetes was increased as well. In this particular study, however, the explanation could be a significantly higher mean age (62.1 ± 8.3 years) in the iNPH cohort compared to ncHC.

Like in many other medical conditions, the occurrence of arterial hypertension and diabetes depends on several factors such as age, race, and gender as well as on the geographic location of the study population. Thus, for example, the prevalence of arterial hypertension could in the same study be found in about 28% of the North American populations and in 44% of the European population, and in addition with a great variation within Europe (ranging from the lowest frequency of 38% in Italy to the highest frequency of 55% in Germany) (Wolf‐Maier et al., 2003). Population‐based studies are therefore required in order to assess the prevalence of these diseases. In the present study, we used data from a big population‐based Norwegian study (The HUNT3 Survey) in order to compare the prevalence of cardiovascular disease and DM with our patient cohorts. As HUNT3 contains data from a typical Norwegian population, the control and source populations are the same, which makes the comparison relevant and reliable.

### 4.5 Limitations

A major limitation of this study was the relatively low number of CMI (*n *= 48) and IIH (*n *= 52) cases, which made statistical comparison with the extremely larger cohort (HUNT3) challenging. Therefore, our biostatistician (A.H.P.) implemented statistical tools suitable for such comparison, as specified in the methods and in the tables. The same statistical analysis was previously performed successfully in similar studies regarding vascular comorbidity in iNPH (Eide & Pripp, 2014) and ncHC (Eide & Pripp, 2016), respectively. However, our observations will still need to be verified in a larger cohort of CMI/IIH patients.

The method used for stating the presence/absence of cardiovascular disease and DM may also be questioned, namely the fact that the presence/absence of the disease was self‐reported.

## 5 Conclusions

This study provided evidence of increased prevalence of DM in male CMI as well as female IIH cases and of AH in female IIH cases. Although requiring further exploration, these findings point to AH and DM as potential risk factors in the pathophysiology of CMI and IIH, as well as to possible common underlying pathophysiology of these two conditions presenting with the reduced ICC.

## Conflicts of Interest

The authors disclose no conflicts of interest relevant for this study.
